# Supplementary material for: Metabolism of Malus halliana Roots Provides Insights into Iron Deficiency Tolerance Mechanisms
Source: Plants (Basel). 2024 Sep 6;13(17):2500. doi: 10.3390/plants13172500 (PMC11397119; doi:10.3390/plants13172500)
Supplement: Supplementary file 1 [file plants-13-02500-s001.zip › plants-3130924-supplementary.pdf]

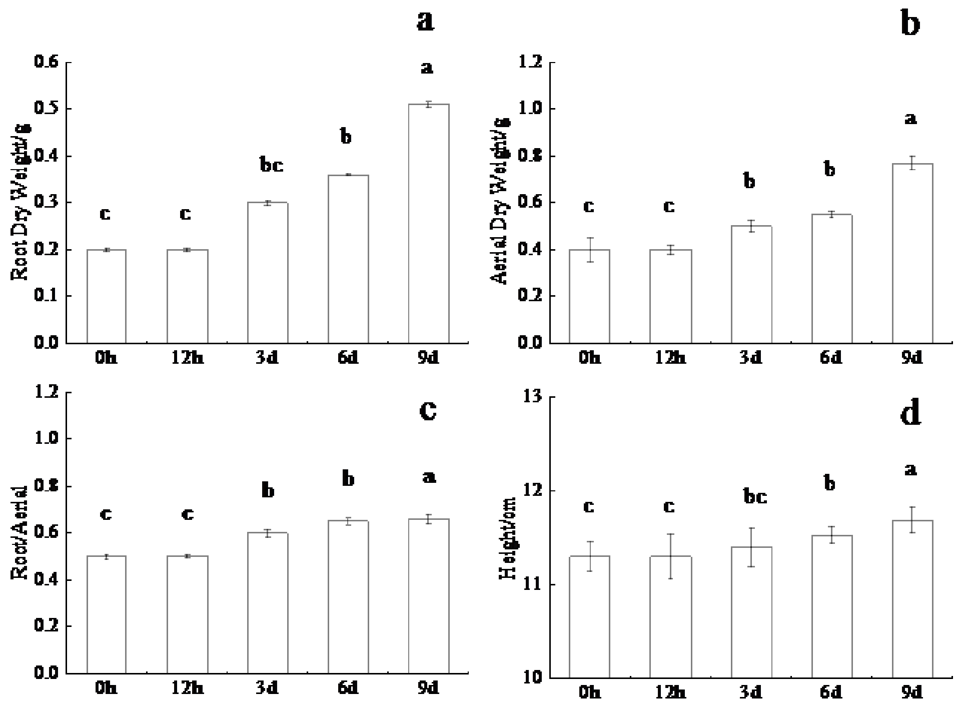

Figure S1. Parameters of *M. halliana* under Fe deficient stress: **a** root dry weight; **b** aerial dry weight; **c** root to aerial; **d** height. Results are shown as mean (n = 3) ± SE. Different letters indicate statistical differences different treatments (One - way ANOVA, $P < 0.05$).

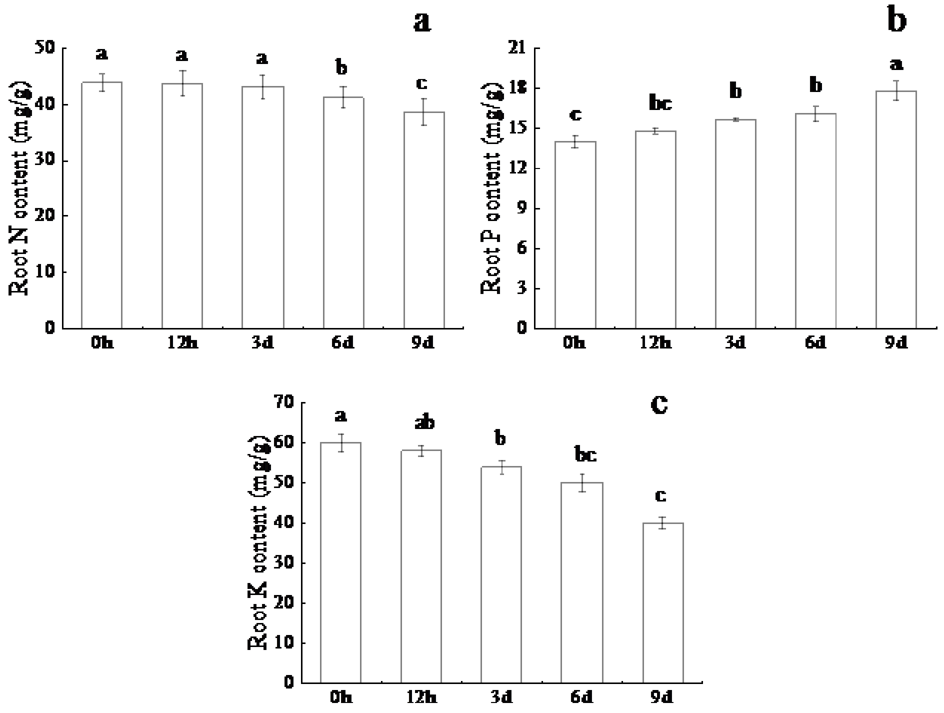

Figure S2. Elemental parameters of *M. halliana* root under Fe deficient stress: **a** nitrogen content;

b phosphorus content; **c** potassium content. Results are shown as mean (n = 3) ± SE. Different letters indicate statistical differences different treatments (One - way ANOVA, $P < 0.05$).

Table S1. Relative concentration and fold-changes in the *M. halliana* of major metabolites under Fe deficient stress.

Timepoint	Metabolites	Quant Mass	VIP	P-value	FC
R12h-R0d	Norleucine	86	2.03691	5.30899E-07	5.03584E-07
	Indolelactate	115	2.03671	7.08557E-07	2.25793E-06
	L-Malic acid	73	1.79917	0.000145402	0.806106889
	adenine	84	1.55173	0.000399918	0.47406363
	3-(4-hydroxyphenyl)propionic acid	179	1.73866	0.000439309	0.094773502
	N-Methyl-DL-alanine	130	1.70165	0.000747707	1.468138763
	aspartic acid	100	1.69377	0.001123272	0.70087648
	phenylalanine	218	1.65382	0.001568429	1.262169528
	N-Methyl-L-glutamic acid	98	1.71889	0.001780634	3.09633E-06
	Methyl Phosphate	135	1.72502	0.001960856	0.503369138
	Maleimide	207	1.6418	0.001991871	1.478553902
	Aminooxyacetic acid	73	1.53478	0.00209883	1.501661018
	oxalic acid	151	1.61555	0.002673869	1.407241796
	Cysteinylglycine	115	1.58659	0.002732845	1.380610864
	fumaric acid	143	1.58653	0.003268495	0.768740118
	3-hydroxy-L-proline	101	1.53691	0.00405438	1.372214814
	Allantoic acid	103	1.53988	0.004763567	1.643053414
	D-Arabitol	103	1.42956	0.005754264	1.651045704
	Citraconic acid degr1	89	1.49375	0.00620982	1.31529380

			1	5
Cumic Acid	221	1.53918	0.006210142	1.333897279
22-Ketocholesterol	204	1.46878	0.006344352	0.617035615
phenylacetaldehyde	105	1.46008	0.007966618	1.326942755
putrescine	102	1.4947	0.008313574	1.329403732
citric acid	147	1.4401	0.008876836	0.760985904
Benzoylformic acid	70	1.46543	0.009473546	0.228226658
1-Methylhydantoin	116	1.44941	0.009631888	1.259000742
dibenzofuran	56	1.44765	0.009704137	1.304583173
p-benzoquinone	79	1.43832	0.010085695	1.317266522
2'-Hydroxyacetophenone	70	1.44549	0.010541262	2922332.717
3-Methylamino-1,2-propanediol	117	1.73854	0.011868225	2.9867884
maltose	204	1.36339	0.012724556	0.763917997
norvaline	221	1.37548	0.013692462	1.323875703
m-cresol	165	1.12374	0.014457096	5.088463765
methyl octanoate	87	1.38493	0.014511489	1.306782552
2-aminoethanethiol	130	1.37547	0.01520672	1.273273971
Epicatechin	73	1.64181	0.015455454	3.113155488
D-Glyceric acid	189	1.40294	0.017461437	0.759544961
D-erythro-sphingosine	204	1.32017	0.018333707	0.492733253
beta-Glutamic acid	100	1.37271	0.019057195	1.570096847
Nicotinoylglycine	207	1.33115	0.02067317	1.248735931

	Methyl Palmitoleate	128	1.3619	0.020740219	0.184191986
	creatine degr	147	1.51197	0.022860826	2.420039023
	xylitol	103	1.2814	0.023007873	1.274522933
	sucrose	73	1.51461	0.024681208	4.187464015
	dl-p-Hydroxyphenyllactic acid	179	1.36035	0.02620692	3.834952192
	kyotorphin	171	1.69721	0.026823828	0.241254574
	Saccharic acid	73	1.32185	0.027771116	0.840046383
	N-Ethylglycine	130	1.27609	0.027964806	1.237522275
	Allo-inositol	217	1.34645	0.027993798	1.434856224
	2-ketoadipate	73	1.31699	0.028652894	0.75004922
	glycine	86	1.28628	0.028790147	1.169675658
	Ethanolamine	100	1.27546	0.03172858	0.808705221
	5-Aminovaleric acid	102	1.25663	0.032440673	1.304564811
	lactic acid	117	1.33889	0.032662797	1.713088765
	Dithioerythritol	221	1.22578	0.033143957	1.309103899
	xylose	103	1.25211	0.033245878	0.70343431
	Methylmalonic acid	147	1.23677	0.034903228	1.286707596
	valine	72	1.24793	0.035320227	0.588111146
	Dodecanol	75	1.20305	0.038409785	1.271261587
	farnesol	103	1.18052	0.042379001	1.274399398
	mannitol	205	1.20313	0.046470613	1.48778957
R3d-R0d	22-Ketocholesterol	204	1.08005	3.79309E-0	0.14823131

			5	2
3-(4-hydroxyphenyl)propionic acid	179	1.68003	5.15391E-05	1.38915E-07
Diglycerol	129	1.54227	8.28298E-05	2.133202219
kyotorphin	171	1.68116	0.000125768	4.87424E-06
phenylalanine	218	1.53902	0.000223202	1.445804545
pyrogallol	77	1.5488	0.00024408	2.014093797
Norleucine	86	1.31622	0.000275104	0.15672961
succinic acid	147	1.54078	0.00031859	1.93251209
Cumic Acid	221	1.51146	0.000510743	1.600284974
Gallic acid	147	1.41862	0.000559509	2.316154408
5-Aminovaleric acid	102	1.51412	0.000599899	1.822829429
Methyl Phosphate	135	1.51932	0.000624189	0.398953271
oxalic acid	151	1.48798	0.000637108	1.757943158
aspartic acid	100	1.45753	0.000679525	0.586033896
Maleimide	207	1.48652	0.000710893	2.312896734
Nicotinoylglycine	207	1.48298	0.000796705	1.947218813
trehalose	73	1.2875	0.00095403	2.409912432
Aminooxyacetic acid	73	1.49276	0.00099623	2.364353657
putrescine	102	1.47836	0.001083921	1.825979303
Indolelactate	115	1.29023	0.001288615	0.221747647
Glucose-1-phosphate	217	1.41612	0.001487692	2.267608622
5-Methoxytryptamine	130	1.46777	0.001502478	1.863225083
D-Glyceric acid	189	1.38847	0.00153209	0.68389762

				4
Dithioerythritol	221	1.44075	0.001566293	1.780132208
Methyl Palmitoleate	128	1.32832	0.001612775	0.000368159
2-aminoethanethiol	130	1.45474	0.001682921	1.825412135
N-Methyl-L-glutamic acid	98	1.38404	0.001780634	3.09633E-06
2-Keto-L-gulonic acid	103	1.28317	0.001838827	0.211743444
N-Ethylglycine	130	1.44803	0.001851864	1.796096192
Pyruvic acid	89	1.28753	0.001884742	2.966826209
dibenzofuran	56	1.43822	0.001911394	1.763069785
p-benzoquinone	79	1.43551	0.001944504	1.765598975
2-ketoadipate	73	1.283	0.001976301	0.249399311
norvaline	221	1.40904	0.002080274	2.081575102
1-Methylhydantoin	116	1.43153	0.002263254	1.682853208
phenylacetaldehyde	105	1.43556	0.002268961	1.69912277
Digalacturonic acid	105	1.43813	0.002282833	2167125.539
Citraconic acid degr1	89	1.41679	0.002540844	1.770652642
Allo-inositol	217	1.36965	0.002684986	1.763631611
Methylmalonic acid	147	1.39698	0.002700687	1.666498727
N-Methyl-DL-alanine	130	1.43338	0.00270264	1.820969919
alanine	116	1.46053	0.003077631	1.936900068
methyl octanoate	87	1.40184	0.003176677	1.746353625
3-hydroxy-L-proline	101	1.40384	0.003405753	1.91234997

Galactinol	81	1.34419	0.003530812	0.461097917
oxamic acid	147	1.40981	0.003735675	1.672832382
ribitol	205	1.29663	0.003807543	1.56842181
Dodecanol	75	1.37526	0.004638001	1.693403269
oxoproline	157	1.36367	0.004811487	2.463579801
2-hydroxypyridine	78	1.16088	0.005937289	1.45022567
methyl trans-cinnamate	57	1.32706	0.006007358	1.914568907
xylitol	103	1.20597	0.006009875	1.508918066
farnesol	103	1.33116	0.006823136	1.662227056
N-Acetyltryptophan	73	1.10199	0.007361123	0.116396411
fructose	103	1.25703	0.007415089	1.575046607
quinic acid	73	1.28111	0.008219269	0.582853126
glycerol	205	1.25122	0.009094603	1.550048411
Tagatose	103	1.22538	0.009549004	1.516738724
L-Malic acid	73	1.23636	0.011706906	0.852157963
Benzyl thiocyanate	91	1.28025	0.011992096	0.245778275
Carnitine	117	1.21133	0.013331108	2.432367779
Bis(2-hydroxypropyl)amine	219	1.26484	0.016225595	0.345949919
valine	72	1.2276	0.01992368	0.554222907
Benzoylformic acid	70	1.03075	0.020849272	0.329636437
threonine	219	1.12354	0.025350158	0.617962997
Lyxose	103	1.07167	0.02633070	1.49400982

				7	7
	creatine degr	147	1.06593	0.028982935	1.362432868
	phloroglucinol	103	1.06642	0.032669954	1.419019411
	lactic acid	117	1.45455	0.042172861	5.420829324
	alpha-ketoglutaric acid	73	1.04762	0.042500754	1.627787841
	asparagine	132	1.1822	0.042664618	0.473988309
	D-erythro-sphingosine	204	1.05907	0.044547234	0.458598201
	urea	69	1.08549	0.048589121	0.458145054
R3d-R12h	succinic acid	147	1.83074	0.000168025	2.354453902
	Diglycerol	129	1.8465	0.000246232	1.782509451
	22-Ketocholesterol	204	1.34582	0.001256264	0.240231371
	Digalacturonic acid	105	1.77466	0.002282833	2167125.539
	2-Keto-L-gulonic acid	103	1.44494	0.002569738	0.267909994
	pyrogallol	77	1.61045	0.003745543	1.595017012
	alanine	116	1.58128	0.005623782	1.779160058
	Pyruvic acid	89	1.49472	0.006088348	2.230395017
	dl-p-Hydroxyphenyllactic acid	179	1.19968	0.006134888	0.052204024
	Nicotinoylglycine	207	1.54228	0.006959707	1.559351953
	myo-inositol	217	1.47369	0.008279454	1.338421262
	Allantoic acid	103	1.45256	0.008475136	0.701802094
	xylose	103	1.45552	0.008587008	1.61410988
	Alizarin	73	1.39065	0.009462161	0.1466681

5-Methoxytryptamine	130	1.46546	0.00992822	1.553712802
Gallic acid	147	1.32654	0.011181343	1.816137009
Maleimide	207	1.48178	0.011805338	1.564296527
Glucose-1-phosphate	217	1.44696	0.013405146	1.711259511
ribitol	205	1.35965	0.013439098	1.488758883
norvaline	221	1.394	0.014993507	1.572334244
Ethanolamine	100	1.35337	0.015095126	1.257475691
Aminooxyacetic acid	73	1.43834	0.015848261	1.574492265
quinic acid	73	1.37131	0.016617369	0.646621342
N-Ethylglycine	130	1.36498	0.018629265	1.451364738
2-ketoadipate	73	1.4595	0.019529721	0.332510593
3-Hydroxypropionic acid	219	1.47235	0.020307439	0.350031831
2-aminoethanethiol	130	1.33051	0.021446189	1.433636575
L-dopa	218	1.48734	0.021997096	0.494785547
glycerol	205	1.31474	0.022156734	1.481624313
Galactinol	81	1.4591	0.024819937	0.553929754
5-Aminovaleric acid	102	1.3025	0.024824753	1.397270119
3-Methylamino-1,2-propanediol	117	1.4918	0.026367832	0.405156293
putrescine	102	1.25094	0.030286236	1.373532554
L-glutamic acid	84	1.17829	0.035378538	1.960625505
1-Methylhydantoin	116	1.22111	0.035685191	1.336657837
dibenzofuran	56	1.19744	0.03932024	1.35144299

			5	1
fumaric acid	143	1.26301	0.04104445	1.311205888
cycloleucine	157	1.06828	0.043555211	0.167572965
benzoic acid	179	1.26592	0.04356776	2.30546733
Dithioerythritol	221	1.17039	0.045094075	1.359809721
Citraconic acid degr1	89	1.15422	0.045125121	1.346203134
p-benzoquinone	79	1.16416	0.045737178	1.340350602
phloroglucinol	103	1.20226	0.047382068	1.451063107
asparagine	132	1.43343	0.048977543	0.452482617
Carnitine	117	1.13873	0.049759413	1.803624138

Table S2 Metabolic pathway in the *M. halliana* under Fe deficient treatment

Timepoints	ID Annotation	Annotation	p-value	-log(p-value)
R12h-R0d	ath01064	Biosynthesis of alkaloids derived from ornithine, lysine and nicotinic acid	5.55E-04	3.26E+00
	ath00630	Glyoxylate and dicarboxylate metabolism	9.99E-04	3.00E+00
	ath00020	Citrate cycle (TCA cycle)	1.05E-03	2.98E+00
	ath01070	Biosynthesis of plant hormones	5.02E-03	2.30E+00
	ath01065	Biosynthesis of alkaloids derived from histidine and purine	5.40E-03	2.27E+00
	ath00480	Glutathione metabolism	6.82E-03	2.17E+00
	ath00330	Arginine and proline metabolism	9.72E-03	2.01E+00
	ath00760	Nicotinate and nicotinamide metabolism	1.03E-02	1.99E+00
	ath00360	Phenylalanine metabolism	1.16E-02	1.94E+00
	ath00310	Lysine degradation	1.23E-02	1.91E+00
	ath01066	Biosynthesis of alkaloids derived from terpenoid and polyketide	1.30E-02	1.89E+00
	ath00260	Glycine, serine and threonine metabolism	1.38E-02	1.86E+00
	ath00500	Starch and sucrose metabolism	1.45E-02	1.84E+00
	ath00040	Pentose and glucuronate interconversions	1.70E-02	1.77E+00

ath00710	Carbon fixation in photosynthetic organisms	2.33E-02	1.63E+00
ath00250	Alanine, aspartate and glutamate metabolism	2.52E-02	1.60E+00
ath00730	Thiamine metabolism	2.93E-02	1.53E+00
ath00910	Nitrogen metabolism	2.93E-02	1.53E+00
ath00770	Pantothenate and CoA biosynthesis	3.15E-02	1.50E+00
ath00970	Aminoacyl-tRNA biosynthesis	4.20E-02	1.38E+00
ath00300	Lysine biosynthesis	4.31E-02	1.37E+00
ath00680	Methane metabolism	4.81E-02	1.32E+00
ath01063	Biosynthesis of alkaloids derived from shikimate pathway	5.33E-02	1.27E+00
ath00640	Propanoate metabolism	5.33E-02	1.27E+00
ath00460	Cyanoamino acid metabolism	6.72E-02	1.17E+00
ath00280	Valine, leucine and isoleucine degradation	6.72E-02	1.17E+00
ath00230	Purine metabolism	6.94E-02	1.16E+00
ath01061	Biosynthesis of phenylpropanoids	7.86E-02	1.10E+00
ath01062	Biosynthesis of terpenoids and steroids	8.05E-02	1.09E+00
ath00240	Pyrimidine metabolism	1.25E-01	9.03E-01
ath00190	Oxidative phosphorylation	1.55E-01	8.10E-01
ath00350	Tyrosine metabolism	1.87E-01	7.28E-01
ath00430	Taurine and hypotaurine metabolism	1.90E-01	7.21E-01
ath00740	Riboflavin metabolism	1.98E-01	7.03E-01
ath00380	Tryptophan metabolism	2.06E-01	6.86E-01
ath00600	Sphingolipid metabolism	2.31E-01	6.36E-01
ath00290	Valine, leucine and isoleucine biosynthesis	2.55E-01	5.93E-01
ath00410	beta-Alanine metabolism	2.78E-01	5.56E-01
ath00620	Pyruvate metabolism	2.86E-01	5.44E-01
ath00030	Pentose phosphate pathway	2.86E-01	5.44E-01
ath00561	Glycerolipid metabolism	2.86E-01	5.44E-01
ath00908	Zeatin biosynthesis	3.37E-01	4.72E-01
ath00650	Butanoate metabolism	3.44E-01	4.63E-01
ath00052	Galactose metabolism	3.51E-01	4.55E-01
ath00340	Histidine metabolism	3.71E-01	4.31E-01
ath00564	Glycerophospholipid metabolism	3.84E-01	4.16E-01
ath00053	Ascorbate and aldarate metabolism	3.91E-01	4.08E-01
ath00051	Fructose and mannose metabolism	3.97E-01	4.01E-01
ath00270	Cysteine and methionine metabolism	4.47E-01	3.50E-01
ath00960	Tropane, piperidine and pyridine alkaloid biosynthesis	5.13E-01	2.90E-01

	ath00966	Glucosinolate biosynthesis	5.43E-01	2.65E-01
	ath00130	Ubiquinone and other terpenoid-quinone biosynthesis	5.53E-01	2.57E-01
	ath00520	Amino sugar and nucleotide sugar metabolism	6.03E-01	2.20E-01
	ath00860	Porphyrin and chlorophyll metabolism	7.40E-01	1.31E-01
L3d-L0d	ath00250	Alanine, aspartate and glutamate metabolism	7.57E-06	5.12E+00
	ath00630	Glyoxylate and dicarboxylate metabolism	1.14E-05	4.94E+00
	ath00020	Citrate cycle (TCA cycle)	8.05E-05	4.09E+00
	ath01064	Biosynthesis of alkaloids derived from ornithine, lysine and nicotinic acid	1.31E-04	3.88E+00
	ath00040	Pentose and glucuronate interconversions	3.94E-04	3.40E+00
	ath01065	Biosynthesis of alkaloids derived from histidine and purine	7.62E-04	3.12E+00
	ath01070	Biosynthesis of plant hormones	1.25E-03	2.90E+00
	ath00052	Galactose metabolism	1.40E-03	2.85E+00
	ath00360	Phenylalanine metabolism	2.16E-03	2.67E+00
	ath00710	Carbon fixation in photosynthetic organisms	2.53E-03	2.60E+00
	ath01066	Biosynthesis of alkaloids derived from terpenoid and polyketide	2.53E-03	2.60E+00
	ath00260	Glycine, serine and threonine metabolism	2.73E-03	2.56E+00
	ath00330	Arginine and proline metabolism	2.89E-03	2.54E+00
	ath00770	Pantothenate and CoA biosynthesis	4.03E-03	2.39E+00
	ath00290	Valine, leucine and isoleucine biosynthesis	4.48E-03	2.35E+00
	ath01061	Biosynthesis of phenylpropanoids	5.96E-03	2.22E+00
	ath00300	Lysine biosynthesis	6.56E-03	2.18E+00
	ath00640	Propanoate metabolism	9.12E-03	2.04E+00
	ath00650	Butanoate metabolism	1.22E-02	1.91E+00
	ath00970	Aminoacyl-tRNA biosynthesis	1.24E-02	1.91E+00
	ath00460	Cyanoamino acid metabolism	1.31E-02	1.88E+00
	ath00760	Nicotinate and nicotinamide metabolism	1.58E-02	1.80E+00
	ath00310	Lysine degradation	1.89E-02	1.72E+00
	ath00430	Taurine and hypotaurine metabolism	2.41E-02	1.62E+00
	ath01063	Biosynthesis of alkaloids derived from shikimate pathway	2.48E-02	1.61E+00
	ath00740	Riboflavin metabolism	2.64E-02	1.58E+00

	ath01062	Biosynthesis of terpenoids and steroids	3.02E-02	1.52E+00
	ath00240	Pyrimidine metabolism	3.43E-02	1.46E+00
	ath00730	Thiamine metabolism	3.93E-02	1.41E+00
	ath00910	Nitrogen metabolism	3.93E-02	1.41E+00
	ath00010	Glycolysis / Gluconeogenesis	5.41E-02	1.27E+00
	ath00030	Pentose phosphate pathway	5.73E-02	1.24E+00
	ath00620	Pyruvate metabolism	5.73E-02	1.24E+00
	ath00660	C5-Branched dibasic acid metabolism	5.73E-02	1.24E+00
	ath00750	Vitamin B6 metabolism	5.73E-02	1.24E+00
	ath00561	Glycerolipid metabolism	5.73E-02	1.24E+00
	ath00380	Tryptophan metabolism	7.49E-02	1.13E+00
	ath00480	Glutathione metabolism	7.76E-02	1.11E+00
	ath00280	Valine, leucine and isoleucine degradation	8.85E-02	1.05E+00
	ath00340	Histidine metabolism	9.98E-02	1.00E+00
	ath00053	Ascorbate and aldarate metabolism	1.12E-01	9.51E-01
	ath00500	Starch and sucrose metabolism	1.24E-01	9.07E-01
	ath00270	Cysteine and methionine metabolism	1.49E-01	8.27E-01
	ath00190	Oxidative phosphorylation	1.79E-01	7.47E-01
	ath00350	Tyrosine metabolism	2.37E-01	6.25E-01
	ath00600	Sphingolipid metabolism	2.65E-01	5.77E-01
	ath00400	Phenylalanine, tyrosine and tryptophan biosynthesis	2.83E-01	5.48E-01
	ath00410	beta-Alanine metabolism	3.18E-01	4.98E-01
	ath00900	Terpenoid backbone biosynthesis	3.35E-01	4.75E-01
	ath00523	Polyketide sugar unit biosynthesis	3.43E-01	4.65E-01
	ath00680	Methane metabolism	3.43E-01	4.65E-01
	ath00960	Tropane, piperidine and pyridine alkaloid biosynthesis	5.70E-01	2.44E-01
	ath00966	Glucosinolate biosynthesis	6.01E-01	2.21E-01
	ath00520	Amino sugar and nucleotide sugar metabolism	6.61E-01	1.80E-01
	ath00230	Purine metabolism	6.82E-01	1.66E-01
	ath00860	Porphyrin and chlorophyll metabolism	7.94E-01	1.00E-01
L3d-L12h	ath00250	Alanine, aspartate and glutamate metabolism	5.56E-05	4.25E+00
	ath00040	Pentose and glucuronate interconversions	1.01E-04	4.00E+00
	ath00052	Galactose metabolism	4.74E-04	3.32E+00
	ath00350	Tyrosine metabolism	5.58E-04	3.25E+00
	ath00020	Citrate cycle (TCA cycle)	7.31E-04	3.14E+00
	ath00360	Phenylalanine metabolism	7.40E-04	3.13E+00

ath01064	Biosynthesis of alkaloids derived from ornithine, lysine and nicotinic acid	3.04E-03	2.52E+00
ath01065	Biosynthesis of alkaloids derived from histidine and purine	3.82E-03	2.42E+00
ath00650	Butanoate metabolism	5.59E-03	2.25E+00
ath00460	Cyanoamino acid metabolism	5.99E-03	2.22E+00
ath00330	Arginine and proline metabolism	6.29E-03	2.20E+00
ath00760	Nicotinate and nicotinamide metabolism	7.31E-03	2.14E+00
ath00310	Lysine degradation	8.78E-03	2.06E+00
ath00190	Oxidative phosphorylation	9.13E-03	2.04E+00
ath01066	Biosynthesis of alkaloids derived from terpenoid and polyketide	9.31E-03	2.03E+00
ath01061	Biosynthesis of phenylpropanoids	1.13E-02	1.95E+00
ath00430	Taurine and hypotaurine metabolism	1.41E-02	1.85E+00
ath00740	Riboflavin metabolism	1.55E-02	1.81E+00
ath01070	Biosynthesis of plant hormones	2.38E-02	1.62E+00
ath00010	Glycolysis / Gluconeogenesis	3.25E-02	1.49E+00
ath01063	Biosynthesis of alkaloids derived from shikimate pathway	3.63E-02	1.44E+00
ath00640	Propanoate metabolism	4.28E-02	1.37E+00
ath01062	Biosynthesis of terpenoids and steroids	6.01E-02	1.22E+00
ath00630	Glyoxylate and dicarboxylate metabolism	6.14E-02	1.21E+00
ath00053	Ascorbate and aldarate metabolism	6.90E-02	1.16E+00
ath00500	Starch and sucrose metabolism	7.68E-02	1.11E+00
ath00380	Tryptophan metabolism	1.71E-01	7.67E-01
ath00520	Amino sugar and nucleotide sugar metabolism	1.91E-01	7.19E-01
ath00710	Carbon fixation in photosynthetic organisms	1.93E-01	7.14E-01
ath00730	Thiamine metabolism	2.15E-01	6.68E-01
ath00910	Nitrogen metabolism	2.15E-01	6.68E-01
ath00770	Pantothenate and CoA biosynthesis	2.22E-01	6.54E-01
ath04070	Phosphatidylinositol signaling system	2.22E-01	6.54E-01
ath00400	Phenylalanine, tyrosine and tryptophan biosynthesis	2.22E-01	6.54E-01
ath00290	Valine, leucine and isoleucine biosynthesis	2.30E-01	6.38E-01
ath00410	beta-Alanine metabolism	2.51E-01	6.00E-01
ath00030	Pentose phosphate pathway	2.58E-01	5.88E-01
ath00620	Pyruvate metabolism	2.58E-01	5.88E-01
ath00660	C5-Branched dibasic acid metabolism	2.58E-01	5.88E-01

ath00750	Vitamin B6 metabolism	2.58E-01	5.88E-01
ath00561	Glycerolipid metabolism	2.58E-01	5.88E-01
ath00300	Lysine biosynthesis	2.58E-01	5.88E-01
ath00900	Terpenoid backbone biosynthesis	2.65E-01	5.77E-01
ath00523	Polyketide sugar unit biosynthesis	2.72E-01	5.65E-01
ath00480	Glutathione metabolism	2.98E-01	5.26E-01
ath00562	Inositol phosphate metabolism	3.05E-01	5.16E-01
ath00564	Glycerophospholipid metabolism	3.49E-01	4.57E-01
ath00260	Glycine, serine and threonine metabolism	3.67E-01	4.35E-01
ath00270	Cysteine and methionine metabolism	4.08E-01	3.89E-01
ath00240	Pyrimidine metabolism	4.24E-01	3.73E-01
ath00960	Tropane, piperidine and pyridine alkaloid biosynthesis	4.71E-01	3.27E-01
ath00970	Aminoacyl-tRNA biosynthesis	5.05E-01	2.97E-01
ath00130	Ubiquinone and other terpenoid-quinone biosynthesis	5.10E-01	2.92E-01
ath00230	Purine metabolism	5.79E-01	2.37E-01
ath00950	Isoquinoline alkaloid biosynthesis	5.87E-01	2.31E-01
